# Non-targeted metabolomic study in plasma in rats with post-traumatic osteoarthritis model

**DOI:** 10.1371/journal.pone.0315708

**Published:** 2025-03-12

**Authors:** Peng-fei Han, Xi-yong Li, Chang-peng Zhang, Chang-sheng Liao, Wei-wei Wang, Yuan Li

**Affiliations:** 1 Department of Orthopaedics, Heping Hospital Affiliated to Changzhi Medical College, Changzhi, Shanxi, China; 2 Department of Orthopaedics, Wenzhou TCM Hospital Of Zhejiang Chinese Medical University, Wenzhou, Zhejiang, China; 3 Department of Graduate School, Graduate Student Department of Changzhi Medical College, Changzhi, Shanxi, China; Fisheries and Oceans Canada, CANADA

## Abstract

**Purpose:**

This study aimed to examine the differential expression profiles of plasma metabolites in rat models of post-traumatic osteoarthritis (PTOA) and elucidate the roles of metabolites and their pathways in the progression of PTOA using bioinformatics analysis.

**Method:**

Plasma samples were collected from 24 SD female rats to model PTOA, and metabolomic assays were conducted. The samples were divided into three groups: the surgically induced mild PTOA group (Group A: 3 weeks postoperative using the modified Hulth model; age 2 months), the surgically induced severe PTOA group (Group B: 5 weeks postoperative using the modified Hulth model; age 2 months), and the normal control group (Group C: healthy rats aged 2 months). Metabolites were structurally identified by comparing the retention times, molecular masses, secondary fragmentation spectra, collision energies, and other metabolite data with a database (provided by Shanghai Applied Protein Technology Co., Ltd.). Target prediction and pathway analysis were subsequently performed using bioinformatics analysis.

**Results:**

The experiment revealed that in the mild PTOA group, levels of Alpha-ketoglutarate, Isocitric acid, Dichloroacetate, and other metabolites increased significantly compared with the normal group, whereas Linolenic acid, Lactose, and others decreased significantly. These findings suggest that these metabolites can serve as biomarkers for the diagnosis of early PTOA. In the severe PTOA group, Diosgenin, Indoleacrylic acid, Alpha-ketoglutarate, Isocitric acid, and others were elevated and may also be used as biomarkers for PTOA diagnosis. Adrenosterone, (+)-chlorpheniramine, and Phenanthridine levels were higher in the severe PTOA group compared to the mild PTOA group, while Menadione, Adenosine 5’-monophosphate, and Arg-Gly-Asp levels were lower.

**Conclusions:**

Taurocholate, indoleacrylic acid, alpha-ketoglutarate, and isocitric acid may serve as biomarkers for PTOA joint injury in rats. Menadione, adenosine 5’-monophosphate, and Arg-Gly-Asp exhibited differential expression between severe and mild PTOA groups in rats, potentially reflecting the injury’s severity. Further investigation into these molecules in human tissues is warranted to ascertain their utility as biomarkers for PTOA in humans.

## 1. Introduction

Post-traumatic osteoarthritis (PTOA) is a joint disease precipitated by year, which has brought a huge and heavy burden to the economy and society [1]. The rising incidence of traffic accidents and high-energy knee injuries has significantly increased the prevalence of PTOA, imposing a substantial economic and societal burden (1). The hallmark of PTOA is the degradation and loss of articular cartilage; however, it is a comprehensive joint disorder that typically involves inflammation, damage to the soft tissues of the joint capsule and synovium, hypertrophic bone alterations, and subchondral bone changes [2]. PTOA often presents with an asymptomatic phase, which hampers early diagnosis and intervention [3]. As the disease advances, treatment options and their effectiveness become limited. Timely detection and diagnosis, however, could facilitate early intervention and potentially halt disease progression [4]. Advancements in research, particularly in the analysis of metabolite expression changes at disease onset, foster optimism that new technologies and multi-omics approaches will enhance future PTOA patient care [5], which is strongly tied to an individual’s trauma history and the accurate and prompt management of said trauma [6].

Metabolomics emerged in the late 20th century, as suggested by Professor Jeremy Nicholson from Imperial College London [7]. Metabolomics, an omics field that followed genomics, transcriptomics, and proteomics, represents an extension of transcriptomics and proteomics, enabling more precise and direct assessments of an organism’s physiological state [8]. Currently, metabolomics is extensively applied across various research domains. By utilizing metabolome technology to analyze metabolic level differences between experimental and control groups, researchers can identify differential metabolites. This approach is instrumental for biomarker discovery and for investigating the biological processes involving these metabolites, including the identification of regulatory enzymes and metabolic pathways [9]. Consequently, increasing attention is being directed toward diverse research areas, such as disease diagnosis, drug target identification, nutrition and health management, personalized medication, plant growth and development, and stress resistance [10]. However, due to variations in study populations and sizes, as well as differences in detection methods and ranges, the results require further validation. Determining how to translate these findings into treatments for PTOA will be the focus of subsequent research.

Our study aimed to differentially screen the expression profiles of various metabolites in plasma from rat models of PTOA. We conducted targeted prediction and pathway analysis using bioinformatics to elucidate the role of metabolites in the development and progression of PTOA.

## 2. Methods

### 2.1. Establishment of animal models and extraction of samples

All experiments and animal care procedures were approved by the Animal Experiment Ethics Committee of Changzhi Medical College (No.: DW2023036). Plasma samples were collected from 24 SD female rats with a PTOA model, and metabolomic assays were conducted. The improved Hulth modeling method was chosen for this modeling. The skin was cut on the inner side of the right posterior knee joint of the rat, and the soft tissue was separated layer by layer into the joint cavity. The medial collateral ligament, anterior cruciate ligament, and the medial meniscus were completely removed, and the incision was sutured layer by layer. The anesthetic (Pentobarbital Merck 25G) was administered intraperitoneally at a dose of 45mg/kg body weight. The samples were divided into three groups: the surgically induced mild PTOA group (Group A: 3 weeks postoperative using the modified Hulth model; age 2 months), the surgically induced severe PTOA group (Group B: 5 weeks postoperative using the modified Hulth model; age 2 months), and the normal control group (Group C: normal rats, age 2 months) [11]. The method of euthanasia for rats is to use high concentration CO2. The container is not pre filled with gas, and CO2 is filled at a rate of 30% to 70% container volume per minute at an equilibrium rate. Post-euthanasia, knee joint cartilage tissue was harvested for tissue specimen preparation. Histological analysis was conducted using hematoxylin-eosin (HE) staining and safranin O fast green staining to assess the pathological grade (Table 1).

**Table 1.** A cartilage histopathology grade assessment-dgrading methodology.

Grade (key feature)	Associated criteria (tissue reaction)
Grade 0: surface intact, cartilage morphology intact	Matrix: normal architecture Cells: intact, appropriate orientation
Grade 1: surface intact	Matrix: superficial zone intact, oedema and/or superficial fibrillation (abrasion), focal superficial matrix condensation Cells: death, proliferation (clusters), hypertrophy, superficial zone Reaction must be more than superficial fibrillation only
Grade 2: surface discontinuity	As above + Matrix discontinuity at superficial zone (deep fibrillation) ± Cationic stain matrix depletion (Safranin O or Toluidine Blue) upper 1/3 of cartilage ± Focal perichondronal increased stain (mid zone) ± Disorientation of chondron columns Cells: death, proliferation (clusters), hypertrophy
Grade 3: vertical fissures (clefts)	As above Matrix vertical fissures into mid zone, branched fissures ±Cationic stain depletion (Safranin O or Toluidine Blue) into lower 2/3 of cartilage (deep zone) ±New collagen formation (polarized light microscopy, Picro Sirius Red stain) Cells: death, regeneration (clusters), hypertrophy, cartilage domains adjacent to fissures
Grade 4: erosion	Cartilage matrix loss: delamination of superficial layer, mid layer cyst formation Excavation: matrix loss superficial layer and mid zone
Grade 5: denudation	Surface: sclerotic bone or reparative tissue including fibrocartilage within denuded surface. Microfracture with repair limited to bone surface
Grade 6: deformation	Bone remodelling (more than osteophyte formation only). Includes: microfracture with fibrocartilaginous and osseous repair extending above the previous surface

### 2.2. Sample preprocessing

The blood was gradually thawed at 4°C and combined with a pre-cooled solution of methanol, acetonitrile, and water (2:2:1, v/v). The mixture was thoroughly vortexed and then sonicated at low temperatures for 30 min, followed by incubation at −20°C for 10 min. After centrifugation at 14,000 g and 4°C for 20 min, the supernatant was vacuum-dried. Subsequently, 100 μL of an acetonitrile-water solution (1:1, v/v) was added for mass spectrometry analysis. The sample was vortexed, centrifuged at 14,000 g and 4°C for 15 min, and the resulting supernatant was subjected to analysis.

### 2.3. Chromatography—mass spectrometry analysis

In this experiment, samples were separated using an Agilent 1290 Infinity LC Ultra Performance Liquid Chromatography System (UHPLC) equipped with a HILIC column. The column temperature was set to 25°C, the flow rate to 0.5 mL/min, and the sample injection volume to 2 μL. The mobile phase consisted of component A: water with 25 mM ammonium acetate and 25 mM ammonia, and component B: acetonitrile. The gradient elution procedure was as follows: from 0 to 0.5 min, 95% B; from 0.5 to 7 min, B linearly decreased from 95% to 65%; from 7 to 8 min, B linearly decreased from 65% to 40%; from 8 to 9 min, B was held at 40%; from 9 to 9.1 min, B linearly increased from 40% to 95%; and from 9.1 to 12 min, B was held at 95%. The injector temperature was maintained at 4°C throughout the analysis. Samples were analyzed in a random sequence to minimize fluctuations in the instrument’s detection signal. System stability and data reliability were monitored by interspersing QC samples within the sample queue.

### 2.4. Q-TOF mass spectrometry conditions

The primary and secondary spectra of the sample were collected using the AB Triple TOF 6600 mass spectrometer after separating the samples by UHPLC. Mass spectrometry was performed with a Triple TOF 6600 mass spectrometer (AB SCIEX) and detected in electrospray ionization (ESI) positive and negative ion modes, respectively. The ESI source setting parameters were as follows: atomized gas auxiliary heating gas 1 (Gas1): 60, auxiliary heating gas 2 (Gas2): The curtain gas (CUR) was set to 30psi, the ion source temperature was set to 600°C, and the spray voltage (ISVF) was set to ± 5500 V (plus and minus modes). Primary mass-to-charge ratio detection range: 60–1000Da, secondary product ion mass-to-charge ratio detection range: 25–1000Da, primary mass spectrometry scanning accumulation time: 0.20 s/spectra, secondary mass spectrometry scanning accumulation time 0.05 s/spectra; The secondary mass spectrum was obtained using data-dependent acquisition mode (IDA), and the peak intensity value screening mode was used, the declustering voltage (DP): ± 60 V (plus and negative modes), the collision energy: 35 ± 15 eV, IDA settings are as follows: Dynamic exclusion of isotope ion range: 4 Da, acquisition of 10 fragment maps per scan.

### 2.5. Data analysis process

The raw data were converted to. mzXML format from Wiff using ProteoWizard. Subsequently, XCMS software performed retention time correction, peak alignment, and peak area extraction. The data underwent preprocessing, metabolite structure identification, and quality evaluation. This research method uses metabolites from the three major metabolic pathways of carbohydrates, proteins, and lipids in the database (provided by Shanghai Applied Protein Technology Co., Ltd.) as the detection catalog. The aim was to identify differentially expressed metabolites, including fatty acids, amino acids, nucleotides, and benzene compounds. Data analysis encompassed univariate and multidimensional statistical analyses, differential metabolite screening, correlation analysis of differential metabolites, and KEGG pathway analysis.

### 2.6. Evaluation of experimental data quality

Comparing the total ion chromatogram (TIC) of QC samples with overlapping spectra, the higher the response intensity and retention time overlap of each peak, the smaller the variation caused by instrument errors throughout the entire experimental process. Perform PCA analysis on the peaks extracted from all experimental and QC samples. The tighter the clustering of QC samples in positive and negative ion modes, the better the reproducibility of the experiment. All metabolites identified in this project (metabolites identified by combining positive and negative ions) are classified and statistically analyzed based on their chemical taxonomy classification information. This study used strict Orthogonal Partial Least Squares Discriminant Analysis (OPLS-DA) VIP > 1 and P value < 0.05 as criteria to screen for differential metabolites, and used multivariate statistical methods such as PCA, OPLS-DA analysis, etc. to perform dimensionality reduction analysis on the collected multidimensional data while retaining the original information to the greatest extent possible. Perform KEGG analysis on all differential metabolites.

In this experiment, we comprehensively evaluated the reproducibility, stability of the experimental instrument, and reliability of the data quality. Overall, the test demonstrated good reproducibility, the analytical system of the experimental instrument was stable, and the data were reliable. The differences in metabolite expression detected in the assay reflect the biological changes inherent to the sample.

### 2.7. Statistical analysis

The statistical software used in this study is SPSS Version 22 (SPSS Inc.). Use Spearman rank correlation analysis to examine the relationship between two sets of data. The result shows the mean SD. In all tests, a P-value less than 0.05 is considered statistically significant.

## 3. Results

### 3.1. Statistics and analysis of appraisal results

#### 3.1.1. Histological analysis and identification.

**Fig 1:**
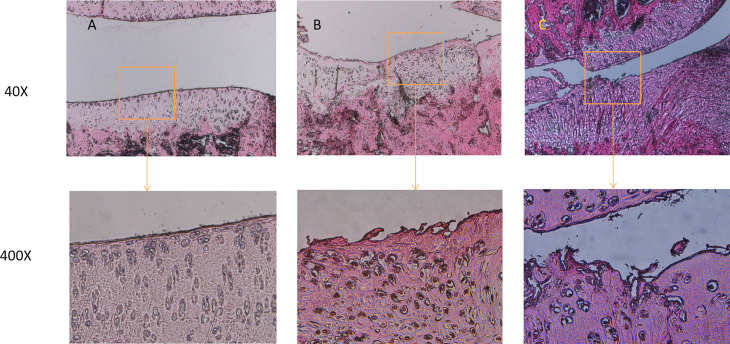
HE staining results of cartilage tissue in different groups of rat PTOA (A: normal group, B: mild PTOA group, C: severe PTOA group).

**Fig 2:**
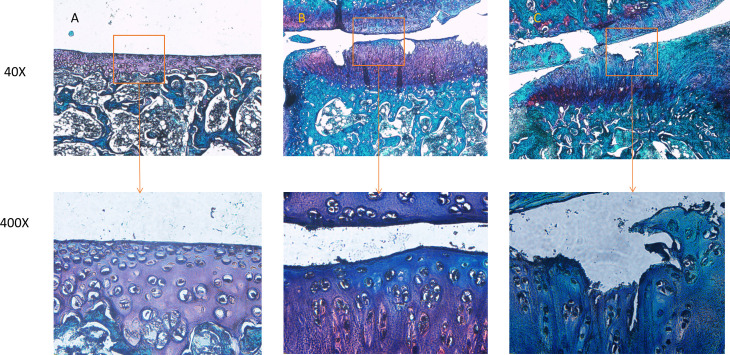
Safranin O fast green staining results of cartilage tissue in different groups of rat PTOA (A: normal group, B: mild PTOA group, C: severe PTOA group).

HE and Safranin O Fast Green staining of cartilage tissue from normal, mild PTOA, and severe PTOA groups in rat knee joints revealed morphological changes. HE staining indicated a progressive decrease in chondrocyte number, increased clustering, and reduced columnar organization as PTOA severity increased. The cartilage surface layer thinned, tissue layers became disordered (superficial, transitional, radial, and calcified layers), and the tide line blurred (Fig 1). Safranin O Fast Green staining yielded similar results (Fig 2). Additionally, we assessed cartilage degeneration on the medial tibial plateau using the OARSI-recommended method, classifying the mild PTOA group as grade 3 and the severe PTOA group as grade 5[12,13].

#### 3.1.2.Metabolite identification quantity and classification statistics.

The metabolite was identified by comparing its molecular mass (with a molecular mass error of < 10 ppm), retention time, secondary fragmentation spectrum, collision energy, and additional data with metabolites in the database (provided by Shanghai Applied Protein Technology Co., Ltd). The identification results were subsequently verified manually on two separate occasions. The assessment level is Level 2 or higher.

**Fig 3:**
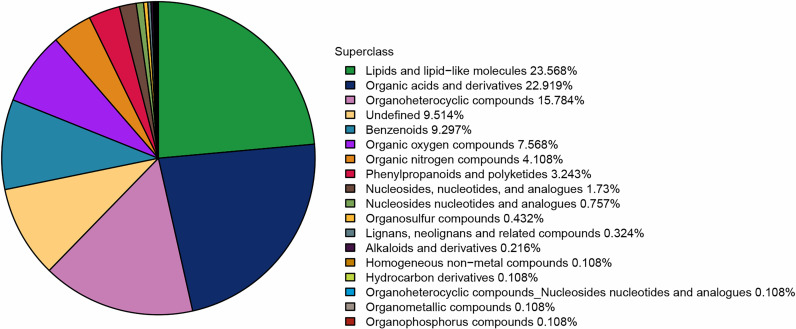
The proportion of identified metabolites in various chemical classifications.

Following the merger of positive and negative ion modes in this project, a total of 925 metabolites were identified. Of these, 587 were detected in the positive ion mode and 338 in the negative ion mode. All identified metabolites, encompassing both positive and negative ions, have been categorized and quantified according to their chemical classification and associated information. Fig 3 depicts the percentage distribution of the various metabolites.

### 3.2. Analysis of inter group differences

Univariate statistical analysis assesses variability within a group and differences between groups for a single variable. In contrast, multidimensional statistical analysis evaluates these aspects across multiple variables. Metabolomic data, characterized by high dimensionality and variable correlation, challenges the effectiveness of traditional univariate analysis in extracting meaningful information. Consequently, employing statistical techniques like PCA, PLS-DA, and OPLS-DA for dimensionality reduction is essential to glean potential insights from complex multidimensional data without compromising the integrity of the original information.

#### 3.2.1. Univariate statistical analysis.

**Fig 4:**
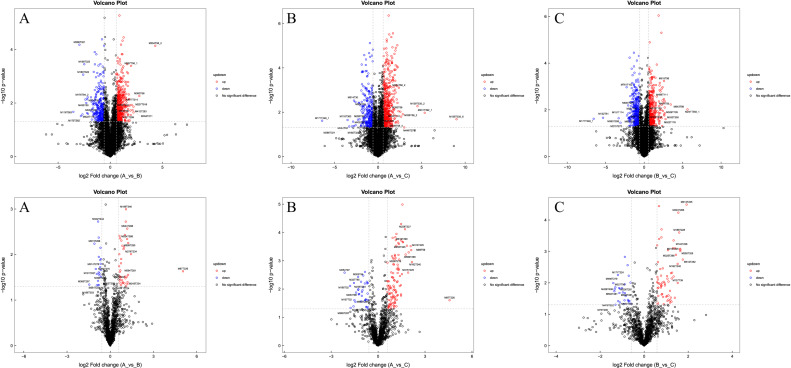
a. Positive ion mode volcanic map (color and up-regulation correlation with differential metabolites) (A) Mild PTOA Group VS Severe PTOA Group (B) Mild PTOA Group VS Normal Group (C) Severe PTOA Group VS Normal Group. b. Negative ion mode volcanic map (correlation between color and up-regulation of differential metabolites) (A) Mild PTOA Group VS Severe PTOA Group (B) Mild PTOA Group VS Normal Group (C) Severe PTOA Group VS Normal Group. The x-axis in the graph shows the logarithmic value of log2 for the fold change, while the y-axis represents the logarithmic value of -log10 for the significance p value. Metabolites that show significant differences are shown in rose red if they meet the criteria of FC >1.5 and p value <0.05. Metabolites that meet the criteria of FC < 0.67 and p value < 0.05 are shown in blue. Metabolites that are not significantly different are shown in black. The marked metabolites in the graph are the ones that show significant differences, and they are labeled with their qualitative names. The top 10 upregulated and top 10 downregulated metabolites are indicated with their respective fold change values (FC).

Univariate statistical analysis is a commonly used method for analyzing data sets. When comparing two samples, popular univariate methods include Fold Change Analysis (FC Analysis) and T-tests/nonparametric tests. These methods help identify significant differences in the data and provide valuable insights for further analysis. Conduct a differential analysis on all identified and unidentified metabolites detected in both positive and negative ion modes based on univariate analysis. Visualize differential metabolites with FC > 1.5 or FC < 0.67 and p-value < 0.05 in the form of volcano plots. For substances with descriptive names that are significantly different, select the top 10 with increased expression changes and the top 10 with decreased expression changes for labeling. Display the results in Fig 4.

#### 3.2.2. Multidimensional statistical analysis.

**Fig 5:**
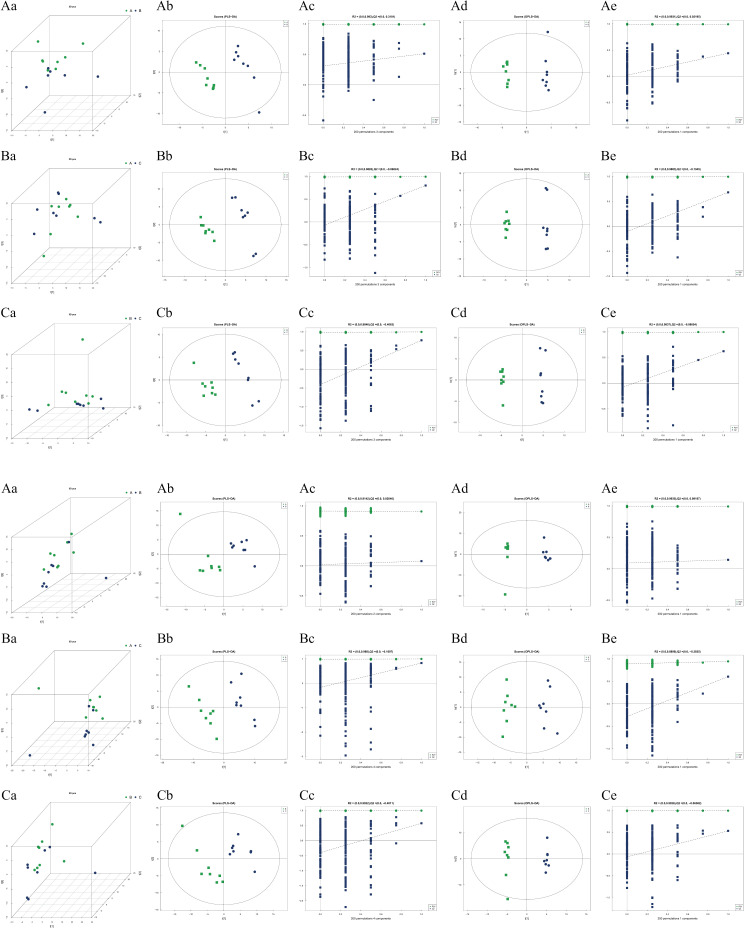
a. Positive ion multi-dimensional analysis chart (A) Mild PTOA group VS Severe PTOA group (B) Mild PTOA group VS normal group (C) Severe PTOA group VS Normal group (a) 3D PCA score chart (b) PLS-DA score chart (c) PLS-DA replacement test (d) OPLS-DA score chart (e) OPLS-DA replacement test. b. Multidimensional analysis of negative ions (A) Mild PTOA group VS Severe PTOA group (B) Mild PTOA group VS normal group (C) Severe PTOA group VS normal group (a) 3D PCA score chart (b) PLS-DA score chart (c) PLS-DA replacement test (d) OPLS-DA score chart (e) OPLS-DA replacement test.

Principal Component Analysis (PCA) linearly combines all metabolites to form new comprehensive variables, achieving dimensionality reduction. The PCA model, obtained through 7-fold cross-validation, demonstrated high cohesion within each group and significant intergroup separation, indicating the model’s reliability and the presence of significant differences between groups. Partial Least Squares Discriminant Analysis (PLS-DA) uses partial least squares regression to relate metabolite expression levels to sample categories, enabling category prediction. Model evaluation parameters from lesions revealed that most models had a Q^2^ greater than 0.5, suggesting stability and reliability. To prevent overfitting in the supervised model, we performed a permutation test. We noted a gradual decrease in both R^2^ and Q^2^ with reduced permutation retention, confirming the absence of overfitting and demonstrating the model’s stability. Orthogonal Partial Least Squares Discriminant Analysis (OPLS-DA), an enhancement of PLS-DA, filters out unrelated noise to improve analytical ability and effectiveness. The OPLS-DA model also showed most Q^2^ values above 0.5, indicating stability and reliability. Permutation tests on each model confirmed no overfitting, and Fig 5 illustrates the model’s robustness.

### 3.3. Screening for differential metabolites

**Fig 6:**
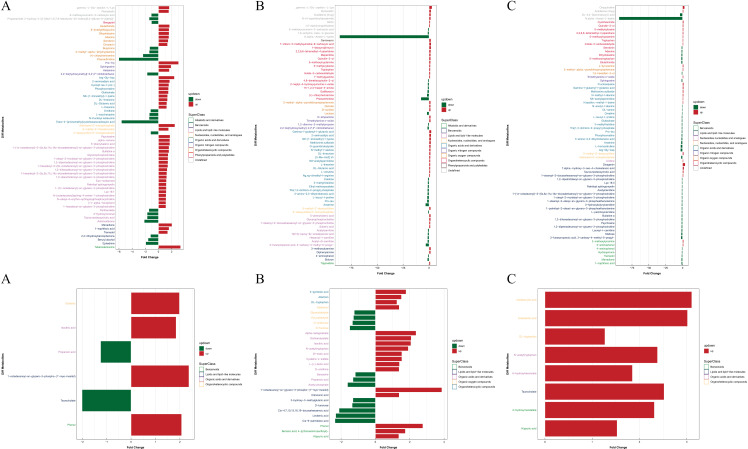
a. Positive ion mode significant difference metabolite expression difference factor analysis (A) mild PTOA group VS severe PTOA group (B) mild PTOA group VS normal group (C) Severe PTOA group VS normal group. b. Negative ion mode significant difference metabolite expression difference factor analysis (A) mild PTOA group VS severe PTOA group (B) mild PTOA group VS normal group (C) Severe PTOA group VS normal group. The abscissa in the figure indicates the difference expression multiple, red indicates that the difference expression multiple is greater than 1, and green indicates that the difference expression multiple is less than 1. Ordinates indicate significantly differential metabolites.

The OPLS-DA model assesses the impact and explanatory capacity of each metabolite’s expression pattern on the classification and discrimination of sample groups using Variable Importance for the Projection (VIP). It identifies biologically significant differential metabolite molecules. Metabolites with an OPLS-DA VIP > 1 and a P value < 0.05 are considered to exhibit significant differences, according to the metabolite screening criteria. A bar chart visually represents the identified significant metabolic alterations, as depicted in Fig 6, Table 2, and Table 3.

**Table 2:** Negative ion mode significant differential metabolites (Fold Change greater than 2 or less than 0.5).

Group		Metabolite name				
A vs B	UP	Phenol	1-octadecanoyl-sn-glycero-3-phospho			
	DOWN	Taurocholate	–			
A vs C	UP	1-octadecanoyl-sn-glycero-3-phospho-(1’-myo-inositol)	Phenol	Alpha-ketoglutarate	Dichloroacetate	Isocitric acid
	DOWN	Cis-9-palmitoleic acid	Linolenic acid	Cis-4,7,10,13,16,19-docosahexaenoic acid		
B vs C	UP	Indoleacrylic acid	Indolelactic acid	Taurocholate	N-Acetyltryptophan	4-Hydroxymandelate
	DOWN	–				

NA: not available

**Table 3:** Positive ion mode significant differential metabolites (Fold Change greater than 2 or less than 0.5).

Group		Metabolite name					
A vs B	UP	Tuberostemonine	Pro-Trp	Adenosine 5’-monophosphate	Arg-Gly-Asp	Menadione	
	DOWN	Phenanthridine	Trans-4-(aminomethyl) cyclohexanecarboxylic acid	(+)-chlorpheniramine	Adrenosterone	–	
A vs C	UP	gamma.-L-Glu-.epsilon.-L-Lys	Gamma-l-glutamyl-l-glutamic acid	2-aminoadipic acid	7-chloro-3-methylquinoline-8-carboxylic acid	N6-(1-iminoethyl)-l-lysine	
	DOWN	N.alpha.-Acetyl-L-lysine	Phenanthridine	Anserine	2-furanpropanoic acid, 3-carboxy-4-methyl-5-propyl-	Lactose	
B vs C	UP	Diosgenin	Oxyquinoline	7.alpha.-hydroxy-3-oxo-4-cholestenoic acid	–		
	DOWN	N.alpha.-Acetyl-L-lysine	DL-2,4-Diaminobutyric acid	Arg-Gly-Asp	L-homocitrulline	2-furanpropanoic acid, 3-carboxy-4-methyl-5-propyl-	Adenosine 5’-monophosphate

NA: not available

In this study, we found that the expression of gamma-L-Glu-epsilon-L-Lys and Gamma-l-glutamyl-l-glutamic acid was significantly upregulated in mild PTOA compared to the normal group. Additionally, 2-aminoadipic acid, N.alpha.- Acetyl-L-lysine, Phenanthridine, and Anserine were significantly down-regulated. In the negative ion mode, 1-octadecanoyl-sn-glycero-3-phospho-(1 ‘-myo -inositol), Phenol, and Alpha-ketoglutarate were also down-regulated. Down-regulated expressions include Cis-9-palmitoleic acid, Linolenic acid, and Linolenic acid. In severe PTOA, compared to the normal group, the expressions of Diosgenin, Oxyquinoline, and 7.alpha.-hydroxy-3-oxo-4-cholestenoic acid were significantly increased, while the expressions of N.alpha.-Acetyl-L, DL-2,4-Diaminobutyric acid, and Arg-Gly-Asp were significantly decreased. In the negative ion mode, the expressions of Indoleacrylic acid, Indolelactic acid, and Taurocholate were significantly up-regulated.

### 3.4. Cluster analysis

**Fig 7:**
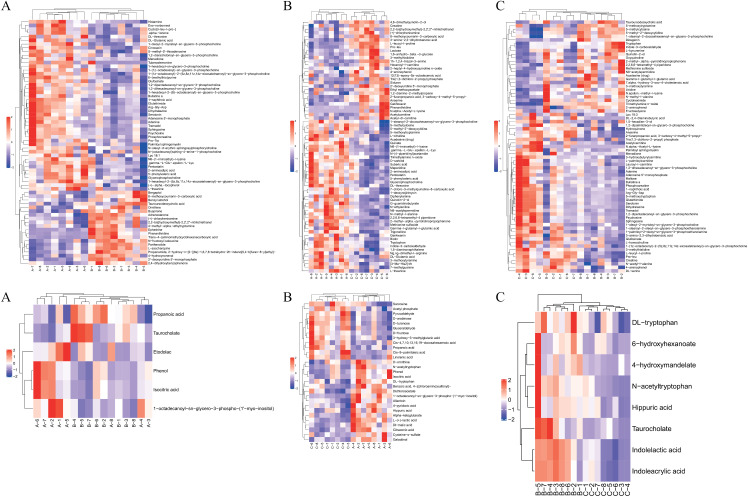
a. Positive ion mode, significant difference, metabolite level clustering heat map (A), mild PTOA group VS severe PTOA group, (B) mild PTOA group VS normal group, (C) severe PTOA group VS normal group. b. Negative ion pattern significant difference metabolite level clustering heat map (A) Mild PTOA group VS severe PTOA group (B) Mild PTOA group VS normal group (C) Severe PTOA group VS normal group. The figure illustrates differential metabolites, with each row representing a specific metabolite showing significant differential expression. The vertical axis depicts the metabolite, while the horizontal axis represents different sample groups. The color red indicates significant upregulation, while blue signifies significant downregulation, with varying shades reflecting the degree of upregulation or downregulation. Similar expression patterns of metabolites are clustered together on the left side of the figure.

To clearly demonstrate the connections and distinctions among the metabolite expression patterns of the samples, we subtracted the group’s average value from the expression levels of all samples and differential metabolites. We standardized the results by dividing them by the root mean square of the group number. Subsequently, we calculated the distance matrix and employed hierarchical clustering for the analysis. The results of the hierarchical cluster analysis for metabolites with significant differences (VIP > 1, p-value < 0.05) are displayed in Fig 7. Metabolites clustered within the same group exhibit similar expression patterns, suggesting potential shared functionalities and involvement in corresponding metabolic processes or cellular pathways.

### 3.5. Correlation analysis

**Fig 8:**
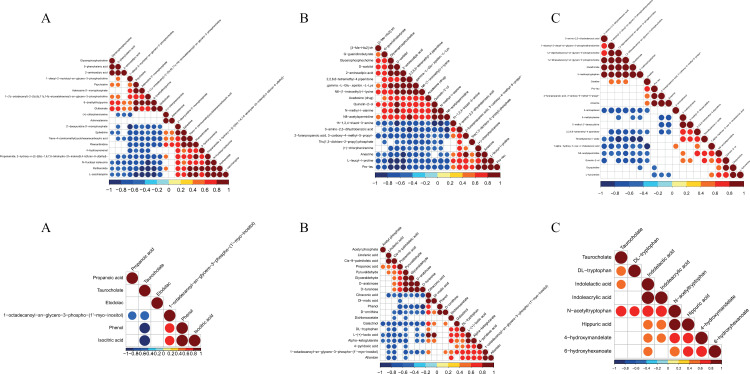
a Positive ion mode correlation heat map (A) Mild PTOA group VS Severe PTOA group (B) Mild PTOA group VS normal group (C) Severe PTOA group VS normal group. b. Negative ion mode correlation heat map (A) Mild PTOA group VS Severe PTOA group (B) Mild PTOA group VS normal group (C) Severe PTOA group VS normal group. In this visualization, the color scheme helps us understand the relationship between variables. Red signifies a positive correlation, blue indicates a negative correlation, and white indicates no significant correlation. The intensity of the color reflects the strength of the correlation coefficient - darker colors represent higher degrees of positive or negative correlation. Additionally, the size of the data points conveys the significance of the correlation. A smaller p-value, indicating higher significance, is represented by smaller data points, whereas a larger point size represents lower significance.

Metabolites with correlated expression may jointly participate in a biological process, termed functional correlation. Moreover, metabolites can interact synergistically or antagonistically. For instance, metabolites exhibiting the same trend of change are positively correlated, while those with opposing trends indicate a negative correlation. Positive correlations may imply a shared synthetic pathway, whereas negative correlations could indicate their use in synthesizing other metabolites. We employed correlation analysis to examine metabolites with significant differences. Using a comparison group as an example, we presented the results through a correlation heatmap in Fig 8.

### 3.6. KEGG Enrichment and Analysis

**Fig 9:**
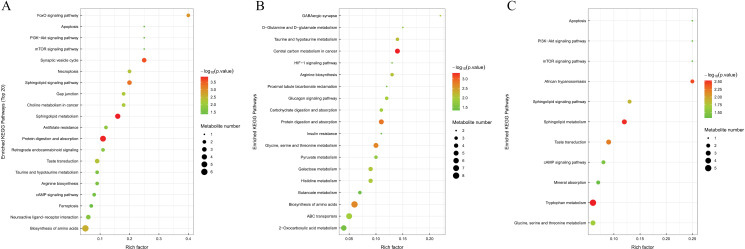
KEGG enrichment pathway chart (bubble chart) (A) mild PTOA group VS severe PTOA group (B) mild PTOA group VS normal group (C) Severe PTOA group VS normal group. In a bubble chart, each bubble represents a metabolic pathway. The selection criteria for these pathways is based on their p-values, with only the top 20 pathways with the highest significance being included. The horizontal placement and size of each bubble in the chart represent the influence of the pathway in topology analysis. The larger the bubble, the greater the influence of the pathway. The vertical placement and color of each bubble represent the p-value of enrichment analysis. The p-values are converted to their negative logarithm (-log10 p-value) and the darker the color, the smaller the p-value. A smaller p-value indicates a higher degree of enrichment and therefore greater significance in the analysis. Furthermore, the rich factor of each pathway represents the proportion of differential metabolites within the pathway, compared to the total number of metabolites annotated in the pathway.

The Kyoto Encyclopedia of Genes and Genomes (KEGG) database is widely used for pathway research. It includes pathway information from various perspectives, such as metabolism, genetic and environmental information processing, cellular processes, biological systems, human diseases, and drug development. Different metabolites interact within organisms to perform biological functions. Analyzing the KEGG pathway offers deeper insights into these functions. To annotate and analyze the KEGG pathway, one must first combine the differential metabolites identified from both positive and negative ion modes. KEGG pathway enrichment analysis employs the KEGG pathway as a unit, considering the metabolic pathways present in the species or those closely related. The study used Fisher’s Exact Test to evaluate and quantify the significance level of metabolite enrichment in each pathway, aiming to identify metabolic and signal transduction pathways significantly affected. A lower P-value indicates a more substantial difference in metabolic pathways. Fig 9 displays the results of the metabolic pathway enrichment analysis.

**Fig 10:**
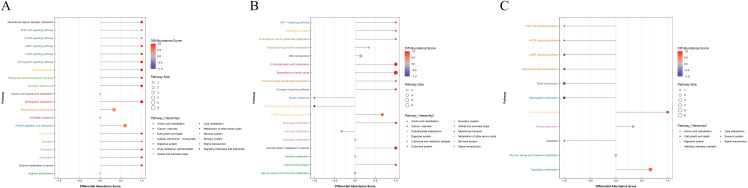
Differential abundance scores of differential metabolic pathways (classified according to Pathway_Hierarchy) (A) mild PTOA group VS severe PTOA group (B) mild PTOA group VS normal group (C) severe PTOA group VS normal group. The figure's Y-axis represents the name of the differential pathway, while the X-axis represents the DA score. The DA score reflects the overall change in all metabolites within the metabolic pathway. A score of 1 indicates an increase in the expression of all identified metabolites in the pathway, while a score of −1 indicates a decrease in their expression. The length of the line segment represents the absolute value of the DA score, and the size of the dot at the end of the line segment indicates the number of metabolites in the pathway. A larger dot signifies a higher number of metabolites. The color intensity of the line segments and dots corresponds to the value of the DA score. The darker red indicates a higher likelihood of overall upregulation in the pathway's expression, whereas the darker blue denotes a higher likelihood of overall downregulation.

Differential Abundance Score is a method for analyzing metabolic changes based on pathways, capturing the average and overall shifts in all metabolites within a specific pathway. Significantly enriched metabolic pathways are classified and attributed according to their higher-level Pathway Hierarchy, as depicted in Fig 10.

## 4. Discussion

Osteoarthritis is a primary contributor to joint disorders, affecting approximately half of the population according to statistical data[14]. The incidence of traffic accidents and sports injuries has recently increased, leading to a rise in cases of traumatic arthritis. This condition results in secondary thickening, hardening, and degeneration of joint cartilage, with the trend showing an annual increase. Traumatic osteoarthritis is a type of osteoarthritis that makes up about 10% of all cases of osteoarthritis[15]. Traumatic osteoarthritis, which accounts for about 10% of all osteoarthritis cases (15), shares some pathophysiological changes with osteoarthritis; however, a complete understanding of their specific pathogenesis remains elusive. Traumatic arthritis typically stems from joint injuries, but its early identification is challenging. Consequently, clinical symptoms fail to improve due to the absence of early intervention.

Metabolomics encompasses the dynamic alterations in metabolites resulting from pathophysiological disturbances, biological stimuli, environmental shifts, and genetic variations. Metabolites not only engage in enzyme-catalyzed chemical reactions but also play crucial roles in cellular functions, including energy production and storage, signal transduction, and apoptosis [16]. Additionally, they serve as both substrates and products of cellular activities. While the host can produce metabolites directly, external factors such as diet and stress can significantly influence their levels [17]. Due to its high sensitivity to initial biological damage, which triggers a cascade of metabolic responses in organisms, metabolomics contains a wealth of information [18]. As a relatively downstream discipline within the “omics” hierarchy, its ultimate aim is to elucidate the collective impact of biological and environmental changes on the profile of low molecular weight molecules in organisms.

In plasma samples, differential metabolites identified by positive ion screening–2-aminoadipic acid, Gamma-l-glutamyl-l-glutamic acid, phenanthridine, and lactose–were differentially expressed between mild PTOA and normal groups. Both 2-aminoadipic acid and Gamma-l-glutamyl-l-glutamic acid showed upregulated expression, while phenanthridine and lactose were downregulated. Differential metabolites alpha-ketoglutarate, isocitric acid, and dichloroacetate, identified by negative ion screening, were upregulated in mild PTOA compared to normal groups. Indoleacrylic acid, indolelactic acid, and taurocholate were differentially expressed in severe PTOA versus normal groups, with all expressions being upregulated.

Studies indicate that elevated cholesterol levels in cartilage cells impede the association with chondrogenesis genes, leading to osteoarthritis[19]. Taurocholate may lower cholesterol in the chondrocyte membrane, thus enhancing the activation of cartilage marker genes and playing a role in OA treatment by promoting chondrocyte proliferation [20]. Our research revealed that taurocholate levels were higher in the severe OA group than in the normal group, suggesting that taurocholate is not only a biomarker for OA diagnosis but also has therapeutic potential. Similarly, indoleacrylic acid and indolelactic acid showed an increasing trend in the severe OA group compared to the normal group. These compounds, typically derived from tryptophan metabolites, have been demonstrated to significantly affect human health and disease[21]. Diosgenin, a steroidal saponin with anti-inflammatory properties, has been shown to reduce mitochondrial oxidative stress damage and apoptosis in OA chondrocytes by activating the JAK2/STAT3 pathway, thereby offering protection to OA chondrocytes[22]. An upward trend of diosgenin was also observed in the severe OA group relative to the normal group.

Preventing excessive oxidation of mitochondrial peroxiredoxin(PRX) is crucial for preserving cellular redox homeostasis and signaling, as observed in chondrocytes in vivo[23]. High oxidation of PRX can impair peroxidase function and increase intracellular ROS levels, contributing to oxidative stress. Menadione has been shown to induce chondrocyte changes through redox cycling, leading to excessive cellular ROS production and subsequent PRX oxidation[24]. This effect is particularly pronounced in older individuals due to their reduced antioxidant capacity[25]. In this study, lower menadione levels were observed in the severe OA group compared to the mild OA group. The tricarboxylic acid cycle, or Krebs cycle, is essential for regulating mitochondrial function and controlling oxidative stress in eukaryotes. Alpha-ketoglutarate and isocitric acid are two key intermediate metabolites in this cycle [26]. Liang Liu et al. found that alpha-ketoglutarate can mitigate oxidative stress in chondrocyte mitochondria and promote chondrocyte autophagy. It can also decrease inflammatory responses in vivo and in vitro, enhance the extracellular matrix, and promote chondrocyte proliferation while inhibiting apoptosis[27]. They suggest that alpha-ketoglutarate is a potential therapeutic target for OA. In our experiment, we also observed that the expression levels of both metabolites in the mild OA group trended upward compared to the normal group. This may represent a self-protective response, enhancing the energy supply by boosting the tricarboxylic acid cycle in OA chondrocytes [28].

## 5. Conclusions

A wide array of metabolites differ in the blood among mild PTOA, severe PTOA, and normal rats. Taurocholate, indoleacrylic acid, alpha-ketoglutarate, and isocitric acid could serve as biomarkers for PTOA diagnosis in rats. Menadione, adenosine 5’-monophosphate, and Arg-Gly-Asp exhibited differential expression between severe and mild PTOA groups in rats, potentially correlating with PTOA severity. These findings may also apply to humans.

## 6. Limitations

However, this study has the following limitations: 1) The study did not directly screen differential miRNAs in human blood. Given the significant individual differences among PTOA patients in clinical practice, this omission could impact the results. In contrast, the rat model allows for better control of variables such as age and weight, yielding more stable outcomes; 2) Instead of identifying a specific metabolite or a few, the study found more than a dozen. This could be due to the small sample size or extraneous factors. Future research with larger sample sizes may refine the findings and yield more precise results; 3) Some outcomes did not align with expectations, which may also be attributable to the limited sample size; 4) The final results require further validation in human blood.
